# Evaluation of Faculty Parental Leave Policies at Medical Schools Ranked by *US News & World Report* in 2020

**DOI:** 10.1001/jamanetworkopen.2022.50954

**Published:** 2023-01-23

**Authors:** Jessica Slostad, Shikha Jain, Marie McKinnon, Sukarn Chokkara, Neda Laiteerapong

**Affiliations:** 1 Division of Hematology-Oncology, Rush University Medical Center, Chicago, Illinois; 2 Division of Hematology-Oncology, University of Illinois, Chicago; 3 Department of Medicine, University of Chicago, Chicago, Illinois; 4 Department of Medicine, Emory University, Atlanta, Georgia

## Abstract

**Importance:**

Physician parents, particularly women, are more likely to experience burnout, poor family-career balance, adverse maternal and fetal outcomes, and stigmatization compared with nonparent colleagues. Because many physicians delay child-rearing due to the rigorous demands of medical training, favorable parental leave policies for faculty physicians are crucial to prevent physician workforce attrition.

**Objective:**

To evaluate paid and unpaid parental leave policies at medical schools ranked by *US News & World Report* in 2020 and identify factors associated with leave policies.

**Design, Setting, and Participants:**

This cross-sectional national study was performed at US medical schools reviewed from December 1, 2019, through May 31, 2020, and February 1 through March 31, 2021, due to the COVID-19 pandemic. All medical schools ranked by *US News & World Report* in 2020 were included.

**Main Outcomes and Measures:**

The primary outcome was the number of weeks of paid and unpaid leave for birth, nonbirth, adoption, and foster care physician parents. Institutional policies for the number of weeks of leave and requirements to use vacation, sick, or disability leave were characterized. Institutional factors were evaluated for association with the duration of paid parental leave using χ^2^ tests.

**Results:**

Among the 90 ranked medical schools, 87 had available data. Sixty-three medical schools (72.4%) had some paid leave for birth mothers, but only 13 (14.9%) offered 12 weeks of fully paid leave. While 11 medical schools (12.6%) offered 12 weeks of full paid leave for nonbirth parents, 38 (43.7%) had no paid leave for nonbirth parents. Adoptive and foster parents had no paid leave in 35 (40.2%) and 65 (74.7%) medical schools, respectively. Median paid parental leave was 4 (IQR, 0-8) weeks for birth parents, 4 (IQR, 0-6) weeks for adoptive parents, 3 (IQR, 0-6) weeks for nonbirth parents, and 0 (IQR, 0-1) weeks for foster parents. About one-third of medical schools required birth mothers to use vacation (29 [33.3%]), sick leave (31 [35.6%]), or short-term disability (9 [10.3%]). Among institutional characteristics, higher ranking (top vs bottom quartile: 30.4% vs 4.0%; *P* = .03) and private designation (private vs public, 23.5% vs 9.4%; *P* < .001) was associated with a higher rate of 12 weeks of paid leave for birth mothers.

**Conclusions and Relevance:**

In this cross-sectional national study of medical schools ranked by *US News & World Report* in 2020, many physician faculty receive no or very limited paid parental leave. The lack of paid parental leave was associated with higher rates of physician burnout and work-life integration dissatisfaction and may further perpetuate sex, racial and ethnic, and socioeconomic disparities in academic medicine.

## Key Points

**Question:**

What are the parental leave policies at the 90 medical schools ranked by *US News & World Report* for birth, nonbirth, adoptive, and foster physician parents?

**Findings:**

In this cross-sectional study, 87 medical schools had available parental leave policies; nearly half provided no paid leave for birth (37 [42%]) or nonbirth (38 [44%]) parents. Similarly, 35 (40%) and 65 (75%) medical schools offered no paid parental leave for adoptive and foster parents, respectively.

**Meaning:**

These findings suggest that leave for birth, nonbirth, foster, and adoptive parents is inadequate for physician parents and likely further contributes to career inequities and burnout.

## Teaser

This cross-sectional study evaluates the parental leave policies at ranked US medical schools, inclusive of birth, adoption, and foster care leave, in the context of other forms of leave.

## Introduction

Physicians often work long hours and have inflexible schedules, leading to greater challenges integrating professional and personal responsibilities.^1^ Becoming a parent can result in stigmatization and discrimination for physician parents, thus many physician parents delay childbearing.^2,3^ Consequently, physicians are more likely to experience infertility and are more likely to consider adoption and foster care as viable options to becoming parents.^3^

Work-life integration dissatisfaction also significantly contributes to burnout.^3,4,5^ Long, inflexible hours along with lack of paid leave impact child care options, which may increase the risk of female physicians or junior faculty reducing work hours or leaving clinical practice altogether. The attrition rate for women in academic medicine further perpetuates the gender gap and bias in leadership, mentorship, and compensation.^5^ Therefore, many physicians who are interested in becoming parents are highly affected by their institutional leave policies.^5,6^

Various studies^7,8^ have assessed parental leave polices but have focused on the top 10 to 20 *US News & World Report* medical schools and cancer hospitals. However, doing so provides a narrow lens of parental leave policies among medical schools that educate only a minority of physicians in the US. Additionally, previous studies^7,8^ have not examined the policies related to adoption or foster care, which are important options for physicians. Parental leave policies can have caveats requiring the use of other leave types (eg, sick, disability) or vacation; these associations with parental leave have not been characterized. Therefore, we evaluated the parental leave policies at ranked medical schools inclusive of birth, adoption, and foster care leave in the context of other forms of leave.

## Methods

### Data Collection

We performed a cross-sectional analysis of the parental leave policies of the 90 medical schools ranked by *US News & World Report* from December 1, 2019, to May 31, 2020. We reviewed pregnancy and parental leave policies for academic faculty on publicly available websites. If the institution did not have publicly available policies (n = 10), we reached out to individual faculty members through our social networks to send electronic copies of the institutional policies and were able to obtain 5 additional institutional policies through this method. Data extraction from leave policies was completed by at least 2 independent researchers (including J.S., M.M., S.C., and N.L.), and discrepancies were resolved by discussion. After data extraction, a verification email was sent to the contact person on the institutional policy (eg, human resources, support services, Office of Academic Affairs) in June 2020 to ensure data accuracy and provide opportunity to respond with corrections. If there was no response from the institution, it was assumed that the investigators’ interpretation was correct. Because of the changing policies due to the COVID-19 pandemic, a second review was completed from February 1 to March 31, 2021. This study received approval from the University of Chicago Biological Sciences Division Institutional Review Board. This study followed the Strengthening the Reporting of Observational Studies in Epidemiology (STROBE) reporting guideline.

In the collected leave policies, we used web search terms including *faculty*, *parental leave*, *maternity leave*, *family leave*, *childrearing*, *child rearing*, *foster care*, *adoption leave*, and *absence*. For each medical school, we recorded the total number of weeks of paid and unpaid parental leave, including any requirements to use vacation leave, sick leave, Family Medical Leave Act of 1993 (FMLA),^9^ and applicability to dual physician parents when listed. We completed this search for birth, nonbirth, adoptive, and foster parents. Additionally, we evaluated institutional factors that may be associated with leave policies, such as private or public hospital designation, ranking quartiles, state paid and unpaid leave policies, and state religiosity and fertility rates.^10,11^

### Statistical Analysis

Data were summarized using descriptive statistics; χ^2^ tests were used to analyze associations between leave policies and institutional characteristics. We explored associations between medical schools having at least 12 weeks of full paid leave for the birth mother and private medical school designation, census region, religiosity, fertility rate, ranking quartile, and state-paid FMLA. A 2-sided *P* value of less than .05 was considered statistically significant. We used R, version 4.1.1 (R Program for Statistical Computing) for analysis.

## Results

There were 90 medical schools ranked by *US News & World Report*, and we attained data from 87 institutions. Among these, 34 (39.1%) were private institutions, and a larger percentage were located in the South (29 [33.3%]) and Northeast (23 [26.4%]) (Table 1). Twenty-seven medical schools (31.0%) were located in states with laws that provide some pay during leave to supplement the federal FMLA, which is unpaid.

**Table 1.** Characteristics of Medical Schools Ranked by *US News & World Report* in 2020^a^

Characteristic	Medical schools (N = 87)
Private	34 (39.1)
Census region	
Midwest	20 (23.0)
Northeast	23 (26.4)
South	29 (33.3)
West	15 (17.2)
Birth parent leave with any pay, wk	
Median (IQR)	6 (0-12)
0	24 (27.6)
1-4	11 (12.6)
5-11	20 (23.0)
12	30 (34.5)
>12	2 (2.3)
Birth parent leave with full pay, wk	
Median (IQR)	4 (0-8)
0	37 (42.5)
1-4	12 (13.8)
5-11	25 (28.7)
12	12 (13.8)
>12	1 (1.1)
Nonbirth parent leave with full pay, wk	
Median (IQR)	3 (0-6)
0	38 (43.7)
1-4	15 (17.2)
5-11	22 (25.3)
12	11 (12.6)
>12	1 (1.1)
Adoption leave with full pay, wk	
Median (IQR)	4 (0-6)
0	35 (40.2)
1-4	16 (18.4)
5-11	23 (26.4)
12	12 (13.8)
>12	1 (1.1)
Foster leave with full pay, wk	
Median (IQR)	0 (0-1)
0	65 (74.7)
1-4	5 (5.7)
5-11	10 (11.5)
12	6 (6.9)
>12	1 (1.1)

^a^ Data are presented as No. (%) of medical schools unless indicated otherwise.

Medical schools differed in their duration and amount of paid leave for birth mothers. Thirty-seven medical schools (42.5%) did not provide birth mother paid leave at full salary, and 24 (27.6%) did not provide any paid leave for birth mothers and required that vacation, sick, or short-term disability leave be used to have any parental leave. Sixty-three medical schools (72.4%) had some paid leave for birth mothers, but only 13 medical schools (14.9%) provided full pay for at least 12 weeks for the birth mother. The median number of weeks of paid leave at full pay for birth mothers was 4 (IQR, 0-8) weeks. Medical schools often required birth mothers to use other benefits during parental leave. About one-third of medicals schools (31 [35.6%]) required birth mothers to use vacation (29 [33.3%]), sick leave (31 [35.6%]), and/or short-term disability (9 [10.3%]). Among these institutions, the majority did not provide any paid leave for birth mothers (0 weeks among 22; 1-11 weeks among 4; and 12 weeks among 5; *P* < .001). Thirteen medical schools (14.9%) required that vacation (4 [4.6%]), sick leave (6 [6.9%]), and/or short-term disability (9 [10.3%]) be used before birth mothers were eligible for leave benefits.

Leave policies were more limited for the nonbirth parent. Nearly half of medical schools did not offer any paid leave for nonbirth parents (38 [43.7%]). When pay was provided for nonbirth parents, it was consistently at the full salary. Only 11 medical schools (12.6%) offered at least 12 weeks of paid leave for nonbirth parents; 37 (42.5%) provided 1 to 11 weeks of paid leave. The median paid leave for nonbirth parents was 3 (IQR, 0-6) weeks. Parental leave for dual physician households was not explicitly included in most policies. Twenty-seven medical schools allowed both parents to take parental leave, but 35 medical schools did not have written policies.

Parental leave for adoption was similarly restrictive. For adoptive parents, 35 medical schools (40.2%) had no paid adoption leave. If paid adoption leave was available, it was provided at full pay. Twelve schools (13.8%) offered 12 weeks of pay, 1 (1.1%) offered more than 12 weeks of adoption leave at full pay, and 39 (44.8%) offered less than 12 weeks of paid leave. The median duration of adoption paid leave was 4 weeks (IQR, 0-6 weeks). Institutions with at least 12 weeks of paid leave for the birth mother (13 [14.9%]) were more likely to also have at least 12 weeks of paid leave for adoptive parents (13 [14.9%]) and nonbirth parents (12 [13.8%]; *P* < .001).

Foster care had the least amount of paid leave and was mentioned in only 27 leave policies (31.0%). For foster parents, 6 medical schools (6.9%) offered 12 weeks of paid leave, while 65 schools (74.7%) offered no paid parental leave. The median duration of paid leave for foster parents was 0 (IQR, 0-1) weeks.

Table 2 provides details on the number of weeks of leave for birth mothers at full pay by institutional factors. Top-quartile medical schools were more likely to have at least 12 weeks of leave at full pay for birth mothers compared with bottom-quartile schools (7 of 23 [30.4%] vs 1 of 25 [4.0%]; *P* = .03). Private medical schools (8 of 34 [23.5%]) were more likely to have at least 12 weeks of full paid birth leave compared with public medical schools (5 of 53 [9.4%]), which was statistically significant (*P* < .001). There were no other significant differences by parental paid leave or in institutional factors for nonbirth or adoptive parents.

**Table 2.** Paid Leave for Birth Mothers by Characteristics of Medical Schools Ranked by *US News & World Report* in 2020^a^

Characteristic	Medical schools (N = 87)					
	Any paid leave, wk			Full paid leave, wk		
	0 (n = 24)	1-11 (n = 31)	≥12 (n = 32)	0 (n = 37)	1-11 (n = 37)	≥12 (n = 13)
Type of institution						
Private	5 (14.7)	13 (38.2)	16 (47.1)	10 (29.4)	16 (47.1)	8 (23.5)
Public	19 (35.8)	18 (34.0)	16 (30.2)	27 (50.9)	21 (39.6)	5 (9.4)
Ranking						
First quartile (highest)	1 (4.4)	9 (39.1)	13 (56.5)	5 (21.7)	11 (47.8)	7 (30.4)
Second quartile	2 (10.0)	11 (55.0)	7 (35.0)	7 (35.0)	12 (60.0)	1 (5.0)
Third quartile	9 (47.4)	5 (26.3)	5 (26.3)	10 (52.6)	5 (26.3)	4 (21.1)
Fourth quartile (lowest)	12 (48.0)	6 (24.0)	7 (28.0)	15 (60.0)	9 (36.0)	1 (4.0)
Census region						
Midwest	5 (25.0)	10 (50.0)	5 (25.0)	5 (25.0)	10 (50.0)	5 (25.0)
Northeast	4 (17.4)	4 (17.4)	15 (65.2)	10 (43.5)	9 (39.1)	4 (17.4)
South	14 (48.3)	12 (41.4)	3 (10.3)	15 (51.7)	11 (37.9)	3 (10.3)
West	1 (6.7)	5 (33.3)	9 (60.0)	7 (46.7)	7 (46.7)	1 (6.7)
State religiosity						
First quartile (highest)	10 (58.8)	5 (29.4)	2 (11.8)	10 (58.8)	5 (29.4)	2 (11.8)
Second quartile	9 (27.3)	18 (54.5)	6 (18.2)	10 (30.3)	19 (57.6)	4 (12.1)
Third quartile	1 (6.7)	4 (26.7)	10 (66.7)	7 (46.7)	5 (33.3)	3 (20.0)
Fourth quartile (lowest)	4 (18.2)	4 (18.2)	14 (63.6)	10 (45.5)	8 (36.4)	4 (18.2)
State fertility						
First quartile (highest)	10 (47.6)	9 (42.9)	2 (9.5)	10 (47.6)	9 (42.9)	2 (9.5)
Second quartile	7 (33.3)	8 (38.1)	6 (28.6)	7 (33.3)	10 (47.6)	4 (19.0)
Third quartile	4 (22.2)	5 (27.8)	9 (50.0)	9 (50.0)	8 (44.4)	1 (5.6)
Fourth quartile (lowest)	3 (11.1)	9 (33.3)	15 (55.6)	11 (40.7)	10 (37.0)	6 (22.2)

^a^ Data are presented as No. (%) of medical schools. Percentages represent row percentages and may not total 100 owing to rounding.

## Discussion

To our knowledge, this cross-sectional study is the most comprehensive review of parental leave policies inclusive of birth parents, nonbirth parents, adoptive parents, and foster parents for physician faculty at medical schools ranked by *US News & World Report*. We found that 27.6% of medical schools did not provide any paid leave for birth mothers and 42.5% provided paid leave at less than full salary. Parental leave policies were even more restrictive for nonbirth, adoptive, and foster parents. Additionally, we found that about one-third of medical schools relied on vacation and sick leave policies to provide birth mothers with parental leave.

Our major finding was that many medical schools did not provide any paid leave to physician birth mothers. Prior studies have evaluated the paid parental leave among the top 12 to 20 medical schools in *US News & World Report*, and median paid leave for birth mothers ranged from 7 to 8 weeks.^7,8^ Other studies defined paid leave based on 12 weeks of pay of any amount.^12^ Our more comprehensive study of all ranked medical schools showed a lower median of 4 weeks of paid parental leave for birth mothers and a lack of any paid parental leave for birth mothers in nearly half of medical schools. Additionally, we found that while 72.4% of medical schools provided some paid leave, only 14.9% provided 12 weeks of leave at full pay. The lack of adequate paid parental leave for birth mothers likely has a large impact on pregnancy and child-rearing decisions for women in academic medicine,^5,6,13^ as well as job satisfaction and attrition.^5,6^ The lack of financial support for birth mothers may partially explain why, despite women accounting for about half of medical school matriculates, women in academic medicine still remain a minority, are less likely to be promoted, are less likely to be in leadership positions, and are paid less compared with their male colleagues.^14,15,16,17^ Furthermore, the duration of paid maternity leave has a significant impact on parents’ mental and physical health and on initiation and duration of breastfeeding.^18^ Adverse effects associated with less than 12 weeks of paid maternal leave include increased rates of depression, maternal ideation of self-harm, early cessation of breastfeeding, mother and infant hospitalization, and infant mortality.^18^ The American Academy of Pediatrics strongly supports 12 weeks of paid parental leave based on the evidence of parental and infant benefits with this duration of leave.^19^ Furthermore, about one-third of medical schools required use of vacation or sick leave to receive any pay during parental leave. By requiring physicians to use sick or vacation leave for parental leave, less time is available for personal or child illness and vacation time, which may perpetuate burnout in physicians. Although there may be a perception that academic female physicians may not want to take 12 weeks of leave, previous surveys have demonstrated that 11 to 12 weeks of leave were most often desired by birth mothers.^6^ For medical schools interested in eliminating the academic gender equity gap, reconsideration of their parental leave policy for birth mothers would be an important first step, especially since 3 months of paid leave is a small financial commitment compared with the cost of replacing a physician (2-3 times their annual salary).^20^

Unfortunately, limited access to paid parental leave also extended to nonbirth parents. Similar to previous studies,^7,8^ we found that the median duration of leave was 3 weeks. Because many early-career physicians consider having children, there are widespread effects of limited paid parental leave in academic medicine for all early career faculty. This problem may be especially challenging for single-parent physicians or physicians from low-income backgrounds. In addition, leave for nonbirth parents has been associated with emotional, psychological, behavioral, and cognitive benefits to the child and increased rates of breastfeeding.^21,22,23^ Thus, the potential financial and health benefits to the birth parent, child, and nonbirth parent are large if longer durations of paid parental leave were available to the nonbirth parent.

Because of the high risk for infertility among physicians, physicians also stand to benefit greatly from adequate adoption and foster care leave policies. Our study unfortunately demonstrated that alternative solutions to becoming parents are not well supported by most medical schools. The insufficient paid leave for adoption and foster care in medical schools impacts many physicians since physicians delay childbirth by about 5 years compared with nonphysicians, which leads to about 1 in 3 to 4 female physicians experiencing infertility.^3^ Furthermore, physicians in procedural specialties are even more likely to be affected by insufficient paid leave because their long training period leads to further delays in childbirth.^2,3^ Explicit and equitable parental leave policies for alternative methods of child-rearing, such as adoption or foster care, would be important to supporting the many academic physicians who have problems with infertility.

When evaluating institutional factors that may help explain paid parental leave policies, medical schools that were in the top quartile of rankings and had private designation were more likely to have 12 weeks of paid leave for birth mothers than schools in the bottom quartile. Medical schools with a higher ranking and private designation may be more able to afford paid leave. While causation is not possible with this cross-sectional study, investment in personnel via paid parental leave may be associated with better employee retention and job satisfaction, which could translate into more sustained research.^18^

### Limitations

This study has some limitations. Although there may be written parental leave policies, the verbiage was often vague regarding the amount of leave promised to employees. There were frequent discrepancies between the independent investigators’ reviews of parental leave policies. Therefore, this could lead to slight variation between actual institutional implementation of leave policies and our investigators’ interpretation of these policies. However, the ambiguous verbiage in many of these policies also highlights the risk of physicians for receiving less leave due to this ambiguity. Policies were often vague or unclear, which would leave room for interpretation. Often, terms such as *primary and secondary caregivers* offered vastly different leave policies. In addition, terminology for parental leave varied by institution and state, such as the use of terms and definitions of *parent*. This can leave families with foster care parents, nonbirth parents, and same-sex couples vulnerable to less paid and unpaid parental leave. In addition, terms such as *primary and secondary caregiver* could play into gender stereotypes and further perpetuate gender disparities and bias.

Many physician parents also may take less leave than promised due to unwritten pressure from administration, medical or surgical departments, colleagues, or financial strain that could not be captured in our data. Additionally, our analysis used the medical schools ranked by *US News & World Report*, thus our findings may not be generalizable to physicians not affiliated with an academic institution. We assumed these policies would apply to same-sex couples, although we could not confirm the applicability of the policies to these families. We also could not evaluate the amount of paid and unpaid leave for dual physician parents. Also, leave policies did not include information related to other important routes to parenthood, including surrogacy and fertility procedures.

## Conclusions

Despite the known benefits of 12 weeks of paid leave for mothers, families, and newborns, many medical schools offer no paid leave, with less paid leave offered to foster care parents. Adequate paid parental leave is crucial to physician well-being; lack of adequate parental leave contributes to worsening burnout and work-life integration dissatisfaction and further perpetuates gender, racial and ethnic, and socioeconomic disparities. The definition of parental leave is expanding, and we call for 12 weeks of paid parental leave with policies that are clear and inclusive at the institutional, regional, and national level.

## Notes

Supplement.
Data Sharing Statement
