# Supplementary material for: Evaluation of Faculty Parental Leave Policies at Medical Schools Ranked by US News & World Report in 2020
Source: JAMA Netw Open. 2023 Jan 23;6(1):e2250954. doi: 10.1001/jamanetworkopen.2022.50954 (PMC9871796; doi:10.1001/jamanetworkopen.2022.50954)
Supplement: Supplement. — Data Sharing Statement [file jamanetwopen-e2250954-s001.pdf]

Data Sharing Statement

Slostad. Evaluation of Faculty Parental Leave Policies at Medical Schools Ranked by US News & World Report in 2020. *JAMA Netw Open*. Published January 23, 2023. doi:10.1001/jamanetworkopen.2022.50954

Data
Data available: Yes
Data types: Data (not involving human participants)
How to access data: The data that support the findings of this study are available from the corresponding author (Jessica_Slostad@rush.edu) upon reasonable request.
When available: With publication

Supporting Documents
Document types: None

Additional Information
Who can access the data: Researchers whose proposed use of data has been approved by corresponding author.
Types of analyses: Data will be made available for any purpose once approved by investigators.
Mechanisms of data availability: Data will be made available with investigator support after approval of a proposal and with a signed data access agreement.
